# Supplementary material for: Synaptic input variation enhances rate coding at the expense of temporal precision in cochlear nucleus neurons
Source: PLoS Biol. 2026 Jan 12;24(1):e3003587. doi: 10.1371/journal.pbio.3003587 (PMC12818756; doi:10.1371/journal.pbio.3003587)
Supplement: Attachment — Submitted filename: R1_Response to Reviewers.pdf [file pbio.3003587.s003.pdf]

We thank the reviewers for their constructive and detailed feedback. Please find our responses in blue below.

Reviewer #1: Summary

This manuscript combines technically demanding in vitro conductance-clamp recordings with computational modeling to investigate how heterogeneity in auditory nerve input strength affects signal encoding in globular bushy cells (GBCs). The authors demonstrate that increasing input variability enhances firing rate but reduces temporal precision, thereby placing GBCs along a functional continuum between coincidence detection and rate coding.

The topic is of high relevance to auditory neuroscience and provides a clear conceptual advance in understanding how morphological variability translates into computational diversity. The study is well designed, the results are internally consistent, and the combination of experimental and modeling approaches is compelling.

Overall, this is a solid and polished study that is nearly ready for publication pending minor revisions aimed at improving language, precision, and contextual discussion.

Thank you for the summary and positive evaluation.

Major Comments

1. Contextualization with prior work:

The discussion would benefit from incorporating additional literature demonstrating convergence-related enhancement of temporal precision. Two relevant studies the authors could consider are:

o Mittring et al., 2023, Brain Stimulation — Showing that convergent optogenetic stimulation of auditory fibers improves temporal precision in bionic sound encoding.
https://www.brainstimjrnl.com/article/S1935-861X(23)01673-X/fulltext

o Strenzke et al., 2009, J. Neurosci. — Demonstrating impaired timing fidelity with reduced presynaptic release (complexin 1 mutation), supporting convergence as a mechanism to improve temporal coding.
https://www.jneurosci.org/content/29/25/7991

Including these will help position the study within a broader context of how convergence and presynaptic mechanisms jointly shape temporal precision in auditory processing.

Thank you for these suggestions. We added the two papers among others to the Discussion section and explained the impact of convergence on improving temporal precision more broadly. As a result, we restructured the Discussion and added the paragraph "Input Heterogeneity as General Concept"

2. Physiological interpretation of input variability:

The Discussion could more explicitly address potential biological origins of the observed

heterogeneity (e.g., developmental variability, experience-dependent plasticity, or differences between low- and high-frequency GBCs).

We added a paragraph "Biological Origin of Input Variation and Methodological Considerations" to the Discussion and discuss these points there.

3. Language and clarity:
While the manuscript reads well, some sentences—especially in the Introduction (lines 51-74) and Discussion—are overly long and could be streamlined for clarity. For example, repeated formulations like "input variation shapes GBC output" could be replaced with more specific phrasing ("increased variance in excitatory conductances enhances firing rate but reduces vector strength").

We revised several sections of the manuscript to improve clarity and readability with particular focus on the sections pointed out by the reviewer.

4. Terminological precision:
Define "temporal precision" early in the Results section in relation to the quantitative metrics used (vector strength, correlation index). Also clarify when "high variation" implies inclusion of suprathreshold inputs versus subthreshold ones.

Thank you for these suggestions.

Clarification of 'temporal Precision': We agree that the term 'temporal precision' can be ambiguous when multiple quantitative metrics are used. In the revised manuscript, we now explicitly refer to the specific quantitative metric used (vector strength or correlation index) when describing results. The term 'temporal precision' is now reserved for broader, conceptual statements about timing fidelity. Suprathreshold inputs: This is an important point and critical for the interpretation of the results. In our experimental design, suprathreshold inputs are not defined explicitly but instead emerged from the gamma distributed input weight sets. To obtain a better understanding of the presence of suprathreshold inputs, we performed an additional analysis quantifying the probability that an individual input triggers an AP. This analysis revealed distinct patterns across variation conditions (see Fig. 1 below):

No variation (v=0): The probability for any single input to trigger a postsynaptic AP was low (<10%), indicating that all inputs were effectively subthreshold

Medium variation (v=0.4): The three largest inputs had a slightly higher, but still modest, probability of triggering an AP, suggesting they remain near but below threshold.

High variation (v=1): The largest input had a high probability (>80%) of triggering an AP, while the second largest remained far less effective (<30%). This indicates that the high-variation condition contains a single functionally suprathreshold input alongside many weaker ones. This is consistent with our computational model in which suprathreshold inputs emerge at variation levels of v>0.7 We added this information to the revised manuscript.

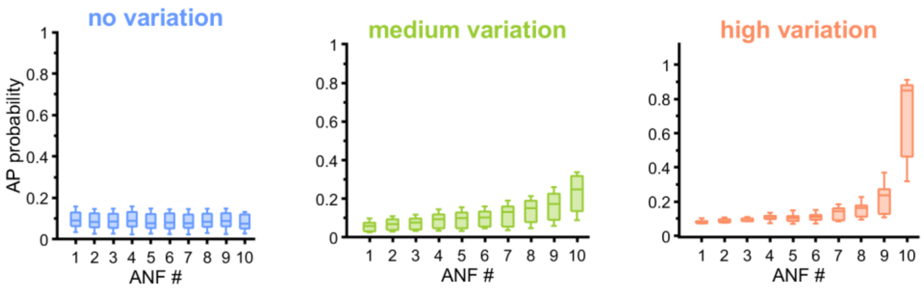

Fig. 1: Probability of individual AN inputs to generate an AP in GBC at different input varations. AN inputs are sorted by increasing synaptic strength.

Minor Comments

* Figures and legends: Please restate sample sizes (n) and the statistical test used in each figure legend for transparency.

Done.

* Abstract: Consider adding one sentence that summarizes the key finding and its trade-off, e.g. "Input heterogeneity enhances rate coding at the expense of temporal precision."

Added.

* Discussion: When referring to downstream processing, briefly note implications for binaural nuclei such as MSO, where BC output patterns are functionally critical.

Globular bushy cells provide the main excitatory drive for the MNTB, which subsequently provides crucial inhibition to key auditory brainstem nuclei, including the MSO and LSO. In the revised manuscript, we now explicitly examine how different input variations modify GBC firing patterns and, consequently, influence the inhibitory signal delivered to these binaural nuclei. We also included a recent publication describing postsynaptic heterogeneity in the MNTB (Chan and Wang, 2025).

* Style and consistency: Maintain consistent tense throughout Methods and Results. Verify that all data and model repository links are accessible.

Thank you for noticing the inconsistency in tense. We have thoroughly reviewed the manuscript, particularly the Methods and Results sections, and ensured consistent use of past tense throughout. A data repository containing all numerical data and model scripts will be made publicly available upon publication at the following identifier: doi.org/10.17632/2tbkzdpjy6/

Strengths

* Excellent integration of electrophysiology and modeling, providing a mechanistic link between structure and function.
* Clear conceptual advance on how input heterogeneity contributes to computational versatility in the auditory brainstem.
* Strong alignment between experimental and modeled results.
* Relevance to broader issues of rate vs. temporal coding in sensory systems.

Weaknesses

* Occasional lack of terminological precision.
* Some overly long or repetitive phrasing that slightly hinders readability.

Reviewer #2: This study examined the impact of the diversity of axonal input strength at auditory nerve - globular bushy cell synapses in gerbil cochlear nucleus in vitro. Based on previous datasets, the authors designed dynamic clamp protocols to simulate realistic activity of these synapses while monitoring output spike probability and timing. The primary conclusion is that when the population of synapses a cell receives varies in strength, the neuron will have an enhanced firing probability (sustained driven rate and spont rate) but at the cost of timing (increased jitter). The results are straightforward and I have only few concerns about the approach and the writing. Overall I would describe the work as a logical extension of the EM/modeling work from Spirou and Manis, but one that brings their conclusions to a more physiological setting.

Specific comments:

Missing information on stimulus paradigm:

1. What is the strength of the single inputs modeled for each of the three input variations conditions? (Fig. 1B plots the ANF strength vs the AND # without scaling. Providing the numbers is essential for reproducibility, thus they should at least scale the y-axis in this figure). It wasn't entirely clear how the values for variation were chosen.

Thank you for pointing this out.

For each cell, the average input strength was first adjusted to fall between 7-14 nS to achieve firing rates of >100 Hz during the 350 Hz stimulation. This average strength was then kept constant for all subsequent recordings. Input strength variations were generated using a gamma distribution, resulting in the following ranges:

- *No variation: all inputs had an equal strength of 7-14 nS*
- *Medium variation: input strengths ranged from 3-6 nS (smallest) to 12-25 nS (largest)*
- *High variation: input strengths ranged from 0.2-0.4 nS (smallest) to 23-46 nS (largest)*

We have added this information to the Methods section and added values to the y-axis of Figure 1B, as suggested.

2. Is the total input strength (i.e., the sum of the ten input) the same across the three conditions? (should be similar, according to the gamma distribution from which the unitary input strength are drawn, but not necessarily the same).

The average input strength was kept constant at 7-14 nS across all variation conditions. Consequently, the total input strength (sum of all ten inputs) was nearly identical: 71 ns for "no variation", 71.0054 for "medium variation", and 70.9988 for "high variation". These minor differences (<0.01%) in total input strengths arise from the discretization in the gamma distribution used to model the inputs. Similarly, the total charge delivered varied by <2% between conditions due to the randomness of ANF discharges. We have added this information to the Methods section of the revised manuscript.

3. Is the stochastic nature of the output solely due to the stimulus tokens or also to that of the postsynaptic properties?

Thank you for that important question. While the stochastic nature of the output is largely influenced by the input spike times, it also depends on the intrinsic properties of the postsynaptic membrane. In an early modeling attempt, we limited the output stochasticity to stem solely from the stimulus tokens, such that the model cell generates APs without inherent temporal jitter. Consequently, the spike timing precision (vector strength and first-spike jitter) was significantly higher in the model compared to the experimental data. Only after introducing a spike-generation jitter of 0.1 ms as an additional source of stochasticity did the model values match the experimental observations. This indicates that inherent spike generation jitter contributes an additional source of stochasticity. This explanation is presented in the Methods section:

"To simulate the intrinsic variability, a spike-generation delay $D_{spike} = D_{fix} + D_{rand}$ was added to each output spike timing. .... This modification was based on the spike latency data from pure-tone stimulation (Fig 3B5 and 3B6) and increased response latency by 0.4 ms (SD = 0.14 ms)."

4. Line 479 authors write that they adjust the mean input strength to a level that elicits a firing rate of about 100 spikes/s in response to a 350 Hz (sound frequency) stimulus. Providing information about these numbers (mean input strength) would allow one to get an idea about the excitability of the neurons, and the variability in excitability. Also, this is an important measure for to enable reproducibility of the study. (this is different in the model, where the mean input strength is a parameter, which they adjusted to match the conductance-clamp data)

We added this information to the Methods section of the revised manuscript and provide mean input strengths and the ranges used for each condition.

5. Authors present each stimulus condition 15 times (e.g., Fig.3B1). Are the conductance stimuli the same 15 across the neurons, or is the conductance stimulus created for each experiment separately?

Across neurons, the same set of conductance stimuli was used. Specifically, the AN input pattern was generated in response to the different sound stimulation and this identical AN spike pattern was applied across variation conditions to ensure that any difference in GBC response was attributable to changes in input variation rather than differences in the stimulus itself. The AN spike pattern and corresponding conductance waveforms for each variation were generated before the experiment and used for all recorded cells. We have now added this clarification to the Methods section.

6. At which inter stimulus interval are the conductance stimuli presented?

Stimuli (duration 200-500 ms) were presented with an identical pause between repetitions. This paradigm was chosen to allow for a sufficient number of spikes per repetition and to mimic in vivo stimulation conditions. This information has been added to the Methods section.

7. Not too important, but: 3 input variations x 15 repeats = 45 stimulations per neuron. Are these 45 stimulations presented in order or are they randomized?

The stimuli were presented in a blocked design, in which all repetitions of one variation were completed before proceeding to the next. This design was chosen for its straightforward implementation, and we controlled for potential time-dependent confounds introduced by the sequential presentation by monitoring key physiological parameters (e.g. membrane potential, AP amplitude) throughout the recordings. Additionally, for a subset of neurons, the initial stimulus condition was repeated at the end of the protocol to assess cell viability. Cells that

exhibited a measurable degradation of vitality were removed from the data set. We added this information to the revised manuscript.

8. Fig. 1C1 (3500 Hz) and Fig. 3B3 show results for the same stimulus, but firing rates that are about 25 - 40 % lower in Fig. 3B3 (calculated from the data in Table 2). Why?

Excellent observation, thank you for pointing this out. The difference in firing rates arises from different analysis windows used for each figure. Fig. 1C1 reports the average firing rate across the entire stimulus duration, including both the onset firing rate and the reduced sustained firing rate. Figure 3B3, in contrast, specifically isolates the sustained firing rate, measured after the onset component of the response. The inclusion of the onset response in Fig. 1C1 therefore produces higher average firing rates compared to the isolated sustained rate shown in Fig. 3B3. When the responses in Fig. 1C1 are restricted to the sustained component, the firing rates are similar to those shown in Fig. 3B3 (see Fig. 2 below). We have clarified this distinction in the revised manuscript.

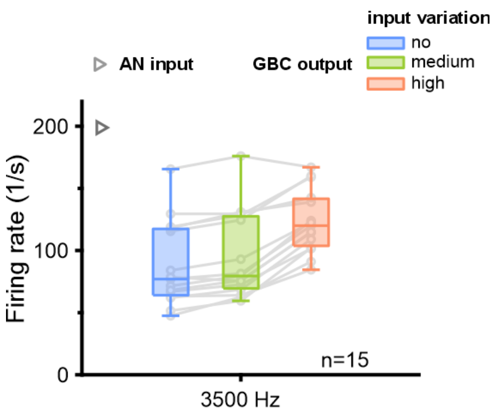

Fig. 2: Firing rates for cells shown in Fig. 1C1 but restricted to the sustained phase of the response.

Discussion:

1. Authors put a lot of emphasis on the contribution of the input variation to temporal and rate coding, but do not consider differences in the excitability of the neurons. Actually, they take steps to reduce differences due to differences in excitability of the neurons (adjusting mean strength of the inputs to match 100 spikes/s). In other words: Can a neuron be tuned to enhance temporal coding by its intrinsic properties (in respect to the GBC population and a given input variation).

Thank you for raising this important point. We agree that from a biophysical standpoint, variations in intrinsic membrane properties, such as input resistance and membrane time constant, can bias a neuron toward temporal or rate coding. However, the primary goal of this study was to isolate the specific contribution of presynaptic variation to sound encoding. By using conductance-clamp to inject conductances based on different input variations, we were

able to systematically assess the impact of input variation independently of the GBC's native synaptic profile, which is unknown at the time of recording.
To address the reviewer's question regarding the potential influence of intrinsic excitability, we calculated input resistance and membrane time constant for a subset of neurons (n = 70/83, 84%). While both properties varied considerably between GBC, they did not correlate with the magnitude of change in firing rate or vector strength between 'no variation' and 'high variation' condition. (Rin vs. firing rate: rs = -0.13, p = 0.68; tau vs firing rates: rs = 0.13; p = 0.67; Rin vs VS: rs = -0.31, p = 0.3; tau vs VS: -0.16, p = 0.59). These results indicate that the functional trade-off driven by input variation operates largely independent of GBC's intrinsic excitability. However, the limited dataset of the current study does not allow us to draw a definitive conclusion, and in the intact circuit, pre- and postsynaptic properties might be co-tuned to further enhance this functional diversity. Future studies combining single-cell physiology with ultrastructural analysis using EM could provide a better understanding of this relationship.
We added this analysis to the results section and extended the Discussion to emphasize this point.

2. Overall the work has a fairly descriptive feel to it: a set of manipulations were done, the results compared, yet while conclusions describe the results, there is no intuitive or biophysical explanation or understanding of why, for example, diversity leads to increased rate and more jitter. It's ironic considering that biophysical analyses are applied here. In this regard the work was a bit uninstructive.

We appreciate the reviewer's critique and agree that explaining the biophysical mechanisms of these results is central to the study's impact. We have significantly revised the Discussion to provide a clear, mechanistic explanation why input diversity alters the trade-off between rate and temporal coding. The biophysical explanation is based on the interaction between synaptic summation and the GBC's action potential threshold. In the low variation condition, with all spikes subthreshold, AP generation relies on the simultaneous arrival of multiple inputs. This coincidence detection filters out uncorrelated noise, resulting in high temporal precision but lower firing rates. Notably, when small inputs are highly synchronized, such as during 350 Hz stimulation, firing rates do not increase further in the high-variation condition. In contrast, high input variation shifts the cell toward rate encoding where large suprathreshold inputs drive the cell independently, bypassing the need for coincidence. This increases the firing rate at the cost of temporal precision. Consequently, the temporal jitter inherent in the largest input is transmitted directly to GBC output, degrading precision. We have rewritten the Discussion section "Computational mechanisms of synaptic convergence" to explicitly address these biophysical principles.

3. Input variation is described as a means to optimize information transfer in the population. Perhaps it is selected for, but it might also be an epiphenomenon of synapse elimination during development, which leads to reduction of some inputs and not others.

Thank you for this interesting thought. Indeed, the true origin of this input variation remains unknown. The morphological reconstruction in the mouse GBC was performed at P60 (Spirou et al., 2023), indicating that this variation is present during adulthood. Whether the variation, however, is a consequence of random synapse elimination during development and how plastic it remains is currently unknown. We now added these considerations to the section "Biological origin of synaptic variation and methodological consideration" in the Discussion.

4. Authors state that half the GBCs operate as coincidence detectors and the other half shows mix-mode operation, citing Spirou. Of course, Spirou's EM paper examined only a tiny fraction of cells and we should not speculate about what the population is like.

We agree with the reviewer. While the work by Spirou et al, was instrumental in motivating the current study, the image volume is naturally limited to only a handful of cells from a single location within the nucleus. Whether this distribution is representative of the entire GBC population and how it may vary between species remains to be investigated. We added this information to the Discussion.

Reviewer #3: This manuscript investigates how the distribution of synaptic strengths onto a type of cell that operates mainly as a temporal coincidence detector influences the temporal patterns of spike generation and the rate of spontaneous activity. Specifically, the work uses a combination of brain slice electrophysiology, with dynamic clamp, and a reduced computational model, to explore this question in the bushy neurons of the gerbil cochlear nucleus. The conclusions are that: 1) when all inputs are sub-threshold and of similar strength, The modeling and experimental data are largely in agreement (there are some exceptions), suggesting that the model captures key mechanisms that control the cell's spiking patterns.

The question is an important and interesting one. The work is inspired by previous anatomical and modeling studies in the cochlear nucleus that have assayed the input terminal sizes (as a proxy for synaptic strength), and recapitulates several of those results, as well as modeling studies that examined combinations of fixed levels of sub-threshold and supra-threshold inputs (Rothman et al 1993). However, it differs from those studies by using statistically-defined nearly

continuous distributions of synaptic strength, rather than either uniform synapses or morphologically-specified synapses from single-cell reconstructions. Importantly, by using dynamic clamp to test the consequences of different input patterns, this work provides a ground truth test of both the reduced model and of some prior modeling predictions. The work uses a reasonable selection of values for the parameter space, and while primarily focussed on 3 domains, results from intermediate distributions are also shown.

The overall conclusion is that the mixing pattern of sub-threshold inputs significantly affects the firing rates. A population bias towards large inputs increases both spontaneous and driven rates, first spike latency jitter, AM rate, and entrainment, while decreasing phase locking synchrony, the correlation index, SAM phase locking.

The paper is very clearly written, well organized and the figures are generally clear.

Thank you for the summary and positive evaluation.

Minor:

line 165: The reference to Ritz and Brownell here seems inappropriate. That study focussed on the PVCN (mostly multipolar and octopus cells), and not on GBCs. Perhaps a different paper was intended?

Thank you for pointing this out. Although Ritz & Brownell describe primary-like responses in the PVCN, they do not link those to bushy cells. We have exchanged the reference by the more appropriate Smith et al, 1991 (PMID: 2022755).

line 179: Did you compute expectation for these "early" spikes based on the spontaneous rate, e.g., using the metric from Chase and Young? This would statistically separate potential spontaneous spikes from spikes that are "first spikes" based on spontaneous rate.

We thank the reviewer for this insightful suggestion. We agree that the differences in spontaneous rates across conditions confound the interpretation of raw first-spike latency by including early spikes that might not be stimulus-driven.
As suggested, we re-analyzed the onset responses using the method described by Chase and Young (2007) which calculates first-spike latency as a statistical deviation from spontaneous activity. Implementing this method, we found that first-spike latency in the 'high variation' condition is now consistently longer, in both the experimental and model data. This suggests that while the large, dominant inputs in the high-variation can depolarize the GBC faster, the increased spontaneous rate obscures the precise onset of a stimulus-evoked response. Notably, when adjusting the previous calculation of "raw" first-spike latencies to exclude spikes triggered

within the first 2 ms of the response (below the first-spike latency of the AN input), the GBC FSL also increases with input variation. We have updated Figures 3 and 5 and the corresponding text in the Results and Discussion section to reflect this new analysis.

465: Just a comment: the idea that the convergence across frequency is limited is also supported to some extend by the Huffman sequence experiments of Carney (1990).

Thank you for pointing this out. We included this reference, as well as the related Wang and Delgutte (2012) paper, in the discussion.

About half the cells associated with GBC show sensitivity to changes in phase spectra, suggesting they receive inputs from AN with different CFs. In our study, we assumed single CF inputs which might be a simplification. We added this clarification to the Discussion section.

Methods; Mention any correction for the junction potential in current clamp, if applied. Otherwise, state "no correction"

For the K-Gluconate based solution, we calculated a liquid junction potential of 12 mV and corrected the reported voltages accordingly in the revised manuscript. This is now explicitly stated in the Methods section.

Figures:
This is really minor, but the unequal widths of the panels in Figures 5 and 6 is a bit confusing. It would make more sense for the model panels to have the same horizontal space as the experimental panels.

Thank you for this suggestion. We agree with the reviewer and adjusted the panels accordingly.

References:

Chan, R. J., and Wang, L.-Y. (2025). Input-specific organization of intrinsic excitability expands coding capacity of fast-spiking auditory neurons. *J. Neurosci.*, e2305242025.

Chase, S. M., and Young, E. D. (2007). First-spike latency information in single neurons increases when referenced to population onset. *Proc. Natl. Acad. Sci. U. S. A.* 104, 5175–5180.

Spirou, G. A., Kersting, M. G., Carr, S., Razzaq, B., Alves-Pinto, C. Y., Dawson, M., et al. (2023). High-resolution volumetric imaging constrains compartmental models to explore synaptic integration and temporal processing by cochlear nucleus globular bushy cells. *Elife* 12, 1–44.

Wang, G. I., and Delgutte, B. (2012). Sensitivity of cochlear nucleus neurons to spatio-temporal changes in auditory nerve activity. *J. Neurophysiol.* 108, 3172–3195.

Xu-Friedman, M. A., and Regehr, W. G. (2005). Dynamic-clamp analysis of the effects of convergence on spike timing. II. Few synaptic inputs. *J. Neurophysiol.* 94, 2526–2534.
